# Prenatal Stress Impairs Spinal Cord Oligodendrocyte Maturation via BDNF Signaling in the Experimental Autoimmune Encephalomyelitis Model of Multiple Sclerosis

**DOI:** 10.1007/s10571-020-01014-x

**Published:** 2020-12-01

**Authors:** Maria Serena Paladini, Davide Marangon, Andrea C. Rossetti, Alice Guidi, Giusy T. Coppolino, Camilla Negri, Vittoria Spero, Maria Pia Abbracchio, Davide Lecca, Raffaella Molteni

**Affiliations:** 1 Department of Medical Biotechnology and Translational Medicine, University of Milan, via Vanvitelli 32, 20129 Milan, Italy; 2 Department of Pharmaceutical Sciences, University of Milan, via Balzaretti 9, 20133 Milan, Italy

**Keywords:** Prenatal stress, Experimental autoimmune encephalomyelitis, Multiple sclerosis, Brain-derived neurotrophic factor, Myelination, Spinal cord

## Abstract

One of the most substantial and established environmental risk factors for neurological and psychiatric disorders is stress exposure, whose detrimental consequences hinge on several variables including time. In this regard the gestational period is known to present an intrinsic vulnerability to environmental insults and thus stressful events during pregnancy can lead to severe consequences on the offspring’s brain development with long-term repercussions throughout adulthood. On this basis, we investigated the long-lasting impact of prenatal stress exposure on the susceptibility to the experimental autoimmune encephalomyelitis (EAE), a well-established murine model of multiple sclerosis. Although stress is considered a triggering factor for this chronic, progressive, autoimmune disease, little is known about the underlying mechanisms. To this end, EAE was induced by immunization with MOG35-55/CFA and pertussis toxin administration in adult female C57BL/6 mice born from control or stressed dams exposed to restraint stress during the last days of gestation. Our results demonstrate that gestational stress induces a marked increase in the severity of EAE symptoms in adulthood. Further, we highlight an altered maturation of oligodendrocytes in the spinal cord of prenatally stressed EAE mice, as indicated by the higher levels of GPR17, a marker of immature oligodendrocyte precursor cells. These behavioral and molecular alterations are paralleled by changes in the expression and signaling of the neurotrophin BDNF, an important mediator of neural plasticity that may contribute to stress-induced impaired remyelination. Since several already marketed drugs are able to modulate BDNF levels, these results pave the way to the possibility of repositioning these drugs in multiple sclerosis.

**Electronic supplementary material:**

The online version of this article (10.1007/s10571-020-01014-x) contains supplementary material, which is available to authorized users.

## Introduction

Stress experience has been consistently established to be a major environmental factor in the etiology of several neurological and psychiatric diseases (Gradus 2017). Thus, living stressful situations can lead to several molecular alterations that can eventually evolve from a normal adaptive body reaction to a medical condition (Davis et al. 2017). This different trajectory may depend on several variables such as the genetic background of the subject, the socio-economic context, the nature, severity and duration of the stressful experience and the time at which the stress occurs. In particular, the gestational period is considered a “window of vulnerability” (Briscoe et al. 2016) and the exposure to adverse events during pregnancy has been shown to impact not only maternal health but also to have a deep long-lasting influence on offspring neurodevelopment, leading to an enhanced susceptibility to diseases and dysfunctions during adulthood (Zucchi et al. 2013; Coe and Lubach 2005; Entringer et al. 2015). It has been hypothesized that, underlying this effect termed “fetal programming” (Barker 1998; Kwon and Kim 2017), prenatal stress (PNS) can leave a signature in the progeny by affecting neural plasticity. In line with this hypothesis, prenatal stress exposure is associated with alterations of the neurotrophin Brain-derived neurotrophic factor (BDNF), a crucial player in neurodevelopment and neuronal plasticity known to be involved in several neurodegenerative and psychiatric diseases (Autry and Monteggia 2012; Zuccato and Cattaneo 2009). For example, PNS has been found to reduce BDNF gene expression in the amygdala and hippocampus of rats at weaning and during adulthood (Boersma et al. 2014), to increase BDNF (exon IV) DNA methylation in the medial prefrontal cortex of adult male rats (Blaze et al. 2017), and to decrease the neurotrophin protein levels in the hippocampus of both female and male rats (Yeh et al. 2012). Of note, changes in BDNF expression were also found in the spinal cord of prenatally stressed adult rats (Winston et al. 2014). Even though the more robust evidences for BDNF modulation by early life stress derive from preclinical studies, it has been also reported in humans that maternal experiences of chronic stress-such as war trauma- are associated with alterations of BDNF methylation in both newborn and maternal tissues (Kertes et al. 2017) and the level of the neurotrophin in the amniotic fluid during pregnancy is positively correlated to maternal early adversity exposure (Deuschle et al. 2018). On these bases, the aim of our study was to investigate the potential long-lasting impact of PNS exposure on the susceptibility to pathologies known to be characterized by alterations of neural function and plasticity, such as multiple sclerosis (MS), an autoimmune disease whose incidence is greatly increasing among young individuals, beginning in adolescence (GBD 2016 Neurology Collaborators 2019). The contribution of environmental factors such as stress in prompting MS or influencing its manifestations and course has not been clearly elucidated (Heesen et al. 2007). Specifically, little is known about the mechanisms by which adverse events during—or around—the gestation period may increase the susceptibility to MS in the progeny. Indeed, to the best of our knowledge, only few clinical studies have linked MS risk with features of maternal behavior including breastfeeding duration (Ragnedda et al. 2015; Conradi et al. 2013), delivery mode (Maghzi et al. 2012; Nielsen et al. 2013) or vitamin D intake (Mirzaei et al. 2011). In this regard, only one report indicating a possible—but not statistically significant—association between stressors, such as late prenatal maternal care and maternal illness during pregnancy and MS has been published so far (Gardener et al. 2009). At preclinical level, most studies using the experimental autoimmune encephalomyelitis (EAE) mouse—the most commonly model for MS—are focused on the long-term effects of neonatal manipulations (Krementsov and Teuscher 2013; Teunis et al. 2002; Columba-Cabezas et al. 2009; Case et al. 2010) but the influence of stress during gestation on EAE has not yet been investigated.

With these premises, the aim of this study has been to evaluate the impact of a prenatal stress exposure on EAE course at adulthood. Specifically, given that MS affects women twice as often as men (Harbo et al. 2013), we induced EAE in the female adult progeny of dams exposed to a stressful manipulation during the last days of gestation and we scored the clinical symptoms in comparison with un-stressed cohorts. Moreover, in order to understand the molecular mechanisms underlying the stress effect, specific molecular analyses have been performed in the spinal cord. In particular, given the demyelinating nature of the encephalomyelitis, we first focused our analyses on markers of different stages of oligodendrocyte maturation. Thereafter, we analyzed the upstream Akt/mTOR signaling pathway, relevant for myelination itself, pinpointing the neurotrophin BDNF as a potential player in the long-lasting influence of PNS on EAE development and MS vulnerability.

## Materials and Methods

### Subjects

Adult female C57BL/6 (*n* = 20) pregnant mice at gestational day (GD) 14 were purchased from a commercial breeder (Charles River Laboratories). Upon arrival, the animals were singly housed with food and water freely available and were maintained on a 12-h light/dark cycle in a constant temperature (22 ± 2 °C) and humidity (50 ± 5%) conditions. All animal experiments were conducted according to the authorization from the Italian Health Ministry (n.1136/2016PR to RM) in full agreement with the Italian legislation on animal experimentation (Italian law DL n. 26, 4th March 2014) and adherent to EU recommendation (EEC Council Directive 2010/63). Accordingly, all the in vivo procedures were carefully refined to minimize animal suffering and to reduce the number of animals used and conducted by staff with certified experience in the use and handling of laboratory animals and in adequately detect any sign of possible discomfort or pain in the animals used. Moreover, the two procedures that may represent a source of suffering for the animals, i.e. the stress exposure and the EAE induction, have been carefully refined as subsequently described. Animal sample size was calculated using the software Piface. The power analysis demonstrated that a sample of 12 mice per group will provide 80% detection power with a 5% type I error. With the inherent variations in mouse behavioral outputs (primary endpoint), we estimated to detect a 30% difference between groups, a standard deviation of 25% and an effect size of *f* = 1.2. For the molecular analyses (second endpoint) we estimated to detect a 15% difference between groups, a standard deviation of 10% and thus an effect size of *f* = 1.5.

### Experimental Conditions and Stress Procedure

**Fig. 1:**
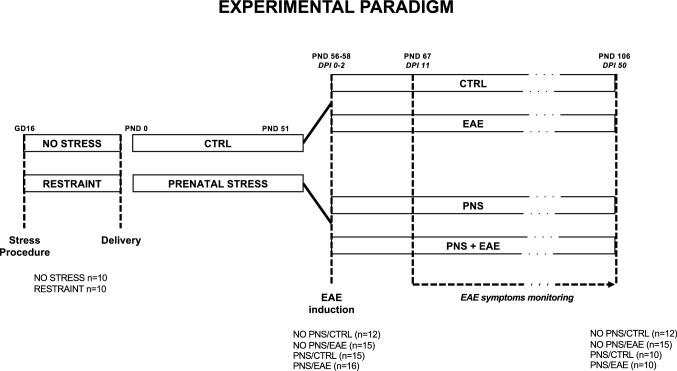
Experimental design scheme. Pregnant female C57BL/6 mice were exposed to daily sessions of physical immobilization stress during the last days of gestation (from GD16 until delivery). Experimental autoimmune encephalomyelitis (EAE) was subsequently induced by immunization with MOG35-55/CFA and pertussis toxin (PTX) administration in the adult (PND 56, DPI 0) female offspring. The effects caused by the previous exposure to prenatal stress (PNS) on EAE progression and severity were then analyzed through the scoring of the EAE clinical symptoms for 50 days (until PND 106, DPI 50)

Ten pregnant dams were randomly selected for exposure to restraint stress, from GD16 until delivery. Briefly, the animals were subjected to multiple daily stress sessions during the last three days of gestation: two sessions at GD16 and GD17 (10.00 a.m. and 14.00 p.m.) and three sessions at GD18 and GD19 (9.30 a.m., 12.30 p.m. and 15.30 p.m.) during which they were placed in plastic cylinders (12 cm long and 4 cm diameter) for 45 min under bright light (3000 lx). Control pregnant females (*n* = 10) were left undisturbed in their home cages. Witnessing stress was limited by blocking any visual and auditory cues, as the stress exposure took place in a different area of the colony room with white noise generators. It is important to underline that this stress protocol does not cause physical damage or severe suffering to the animal, as demonstrated by the absence of differences between the stressed and the control dams regarding the number of delivered pups or their aptitude for maternal care (see “Results” section). Indeed, the aim of the stress exposure was to reproduce a mild and subthreshold discomfort that could represent a vulnerability factor for subsequent stimuli. All pregnant females gave birth except for 2 non stressed dams. At weaning (post-natal day 21, PND21) female pups from control and stressed mothers were subjected to one of four experimental conditions: (i) animals born from non-stressed dams NO PNS/CTRL (*n* = 12), (ii) animals born from non-stressed dams and exposed to EAE induction NO PNS/EAE (*n* = 15), (iii) animals born from stressed dams PNS/CTRL (*n* = 15), (iv) animals born from stressed dams and exposed to EAE induction PNS/EAE (*n* = 16). All animals were socially housed with non-littermates (*n* = 4 per cage) under standard laboratory conditions. A graphic representation of the experimental paradigm can be found in Fig. 1.

### Nest Building Test

The nest building test was performed to investigate changes in the well-being of the animals. Raw material (nesting paper) was provided to all the pregnant females and, the day after the birth, the complexity of the nest has been scored by four different blind operators according to the following scale: 0 = the animal has not manipulated the building material; 1 = the material has been manipulated but the position of the nest is not clear (sparse paper on the bottom of the cage); 2 = flat nest, without vertical walls; 3 = "cup" nest with walls less than half the height of a nest with a full dome; 4 = nest with incomplete dome; 5 = nest with full dome.

### Experimental Autoimmune Encephalomyelitis (EAE)

EAE was induced in 8-week-old female by subcutaneous immunization in the flanks and at the base of the tail with 300 μg of myelin oligodendrocyte glycoprotein (MOG35-55, Espikem #EPK1) per mouse in Incomplete Freund's adjuvant (IFA, Sigma Aldrich #F5506) supplemented with 8 mg/ml of Mycobacterium tuberculosis (strain H37Ra, Difco #231141). Mice immunized received 500 ng of pertussis toxin (PTX, Duotech #PT.181) intravenously the day of the immunization and 48 h later. Animals were daily (11 a.m.) weighted and scored for clinical symptoms of EAE according to the standard EAE scoring system: 0 = healthy, 1 = flaccid tail, 2 = ataxia and/or paresis of hindlimbs, 3 = paralysis of hindlimbs and/or paresis of forelimbs, 4 = tetraparalysis, 5 = moribund or death (Furlan et al. 2009), by two blind operators. Symptoms onset, maximum score (Cs max) and cumulative disease score (sum of daily clinical scores of each individual mouse, CDS) were analyzed. Non-EAE controls received PTX injections, as well as the initial injections of emulsion but without the encephalitogen, to ensure that observed effects are due to EAE and not to reactions to the ancillary components used to facilitate disease induction. In order to avoid suffering related to poor food intake, when the first signs of weakness or paralysis of the limbs appear, food was made available in the cage and the amount of bedding was increased in order to allow animals to reach the water dispenser without any difficulty. During the experiment, a total of *n* = 3 (1 NO PNS/EAE and 2 PNS/EAE) mice died due to the severity of the EAE symptoms. At PND 106 (i.e. day post injection 50), one animal at a time was moved to a separate room to be quickly sacrificed by cervical dislocation, the spinal cord was dissected, frozen on dry ice and stored at − 80° until molecular analyses.

### RNA Preparation and Gene Expression Analyses

For gene expression analyses, total RNA was isolated from the whole cervical portion of the spinal cord by single step guanidinium isothiocyanate/phenol extraction using PureZol RNA isolation reagent (Bio-Rad Laboratories S.r.l., #7326880) according to the manufacturer’s instructions and quantified by spectrophotometric analysis. The samples were then processed for real-time polymerase chain reaction (PCR) as previously reported (Rossetti et al. 2018) to assess mRNA levels of: BDNF total form, BDNF 3′ UTR long, BDNF exon IV and BDNF exon VI. Briefly, an aliquot of each sample was treated with DNAse to avoid DNA contamination and subsequently analyzed by TaqMan qRT-PCR instrument (CFX384 real-time system, Bio-Rad Laboratories S.r.l.) using the iScript one-step RT-PCR kit for probes (Bio-Rad Laboratories S.r.l., #1725141). Samples were run in 384-well format in triplicate as multiplexed reactions with a normalizing internal control (β-actin). Thermal cycling was initiated with incubation at 50 °C for 10 min (RNA retrotranscription), and then at 95 °C for 5 min (TaqMan polymerase activation). After this initial step, 39 cycles of PCR were performed. Each PCR cycle consisted of heating the samples at 95 °C for 10 s to enable the melting process, and then for 30 s at 60 °C for the annealing and extension reactions. A comparative cycle threshold (Ct) method was used to calculate the relative target gene expression versus the control group. Specifically, fold change for each target gene relative to β-actin was determined by the 2^− Δ(ΔCT)^ method, where ΔCT = CT target-CT β-actin and Δ(ΔCT) = CT exp. group-CT control group and CT is the threshold cycle. For graphical clarity, the obtained data were then expressed as percentage versus control group, which has been set at 100%.

### Protein Extraction and Western Blot Analyses

Whole cervical spinal cord samples were manually homogenized using a glass-glass potter in a pH 7.4 cold buffer (containing 0.32 M sucrose, 1 mM MgCl_2_, 1 mM NaHCO_3_, 10 mM HEPES solution, and 0.1 mM phenylmethylsulfonyfluoride in presence of a complete set of proteases [Roche, # 11836145001] and phosphatase [Sigma-Aldrich, # P5726] inhibitors) and then sonicated for 10 s at a maximum power of 10% to 15% (Bandelin Sonoplus). The homogenate was clarified (1000 g; 10 min) obtaining a pellet (P1) enriched in nuclear components, which was resuspended in a buffer (20 mM HEPES, 0.1 mM dithiothreitol, 0.1 mM EGTA) supplemented with protease and phosphatase inhibitors. The supernatant (S1) was then centrifuged (13,000×*g*; 15 min) to obtain a clarified fraction of cytosolic proteins (S2). The pellet (P2), corresponding to the crude membrane fraction, was resuspended in the same buffer used for the nuclear fraction. Total protein content was measured according to the Bradford Protein Assay procedure (Bio-Rad Laboratories), using bovine serum albumin (BSA) as calibration standard.

Protein analyses were performed in the whole homogenate (targeting mature BDNF, phospho-mTOR at Ser2448, mTOR), in the cytosolic fraction (targeting phospho-Akt at Ser473 and Akt), and in the crude membrane fraction (targeting MAG) of 6 mice/experimental conditions. Equal amounts of protein (12 or 16 μg) were run under reducing conditions on polyacrylamide gels and then electrophoretically transferred onto nitrocellulose membranes. Unspecific binding sites were blocked with 10% nonfat dry milk; then the membranes were incubated overnight with the primary antibodies and for 1.5 h at room temperature with a peroxidase-conjugated anti-rabbit or anti-mouse IgG. Specifically, the following primary antibody dilutions were used: against mature BDNF (Icosagen, cod: 327-100) 1:500 in 3% nonfat dry milk; phospho-mTOR/mTOR (Cell Signaling Technology, cod: #2971, #2972) 1:1000 in BSA 5%; phospho-Akt/Akt (Cell Signaling Technology, cod: #9271, #9272) 1:1000 in BSA 5%; MAG (Cell signaling Technology, cod: #9043) 1:1000 in 5% non-fat dry milk and β-actin (Sigma-Aldrich, #A5316) 1:20,000 in 3% non-fat dry milk. All the primary antibodies utilized were validated for western blot by the suppliers. Immunocomplexes were visualized by chemiluminescence using the ECL ETA C 2.0 (Cyanagen #XLS070) or ECL SUN (Cyanagen, #XLS063). Results were standardized using β-actin as the internal control, which was detected by evaluating the band density at 43 kDa. Protein levels were calculated by measuring the optical density of the immunocomplexes using chemiluminescence (Chemidoc MP Imaging System, Bio-Rad Laboratories (imagelab)). To ensure that autoradiographic bands would be in the linear range of intensity, different exposure times were used.

### Immunofluorescence Staining and Cell Count

Three animals from each condition that displayed clinical scores representative of their experimental group were dedicated to immunofluorescence staining. Mice were anesthetized with ketamine (100 mg/kg) and xylazine (10 mg/kg) and perfused transcardially with 0.1 M EDTA (Sigma Aldrich, #03,620) in saline followed by 4% neutral buffered formalin (Sigma Aldrich, #1004960700) in deionized water. Spinal cords were collected and post-fixed for 1 h in the same solution at 4 °C, cryoprotected in 30% sucrose for 24 h, embedded in OCT and then frozen at -80 °C. Spinal cords were cut transversally into 20 μm-thick sections with a cryostat and processed for immunofluorescence. Slides were incubated for 45 min at room temperature with a blocking solution composed by 10% goat normal serum (Sigma Aldrich, #G9023) and 0.1% triton X-100 in phosphate buffered saline (PBS). Then, the sections were incubated with rabbit polyclonal anti-GPR17 (1:2500, custom antibody produced by PRIMM) overnight at 4 °C in PBS with 5% goat normal serum and 0.1% Triton X-100. Following primary antibody incubation, the sections were washed and incubated with the biotinylated secondary antibody (Vector Labs, #BA-1000) for 1 h at room temperature. GPR17 labeling was detected with the high sensitivity tyramide signal amplification kit (Perkin Elmer, #NEL700001KT) according to the manufacturer’s instruction. Hoechst 33,528 was used to visualize cell nuclei. After processing, sections were mounted on microscope slides with fluorescent mounting medium (Dako, #S3023). The custom anti-GPR17 primary antibody used in this publication has been validated (Coppolino et al. 2018) and can be obtained by academic researchers upon request.

For each animal 3 sections from the cervical spinal cord (C1–C8 range) were entirely reconstructed with Adobe Photoshop CC following acquisition of 8–10 images at 10× magnification for each section. GPR17 positive cells were counted manually with ImageJ software in the whole white matter of each section reconstructed and then normalized to the relative area.

### Study Design and Statistical Analysis

This study was not pre-registered. Mice were randomized into the experimental groups (stratified randomization). Biochemical assays were performed using blinding codes and counterbalancing to ensure that treated and untreated conditions appear in adjacent lanes to mitigate regional assay variance (e.g. gel edge effects). All the molecular analyses were repeated 2/3 times and the results confirmed. Analyses of body-weight gain—of the mothers and of the pups before and after the EAE induction—and EAE clinical score were performed with Two-way analysis of variance (ANOVA) with repeated measures (RM). Sphericity was not assumed and Geisser-Greenhouse correction was used. Comparisons between stressed animals or non-stressed mice in nest building assessment test score, number of pups, ratio between female and male newborn, EAE cumulative disease score (CDS), maximum score (CS max) and day of EAE onset were analyzed by two-tailed Unpaired *t* test. The effects of PNS and EAE on the mRNA or protein levels of the molecular targets were conducted with a Two-way ANOVA followed—when appropriate—by a Single Contrast Post Hoc Test (Fisher’s Protected LSD). Differences in the GPR17 positive cell count and MAG protein levels were evaluated using two-tailed Unpaired *t* test. The Shapiro–Wilk normality test was performed to analyze the data distribution for each group. Homogeneity of variance was evaluated using Brown–Forsythe and *F* test for ANOVA and Unpaired *t* test analyses respectively. All the data presented in the manuscript passed both tests and were analyzed as normally distributed and with equal variances. All the molecular analyses were carried out in individual animals (independent determinations) and for graphic clarity, data are presented as means percent ± standard error (SEM) of control group, with significance threshold set at *P* < 0.05. We removed EAE mice that did not show symptoms at day post injection 50 from the molecular analyses (NO PNS/EAE *n* = 4; PNS/EAE *n* = 4). Otherwise, outliers that were more than two standard deviations away from the mean were removed, as they likely resulted from technical errors.

## Results

### Behavioral Analyses

#### Impact of Stress Exposure on Dams and Female Offspring

**Fig. 2:**
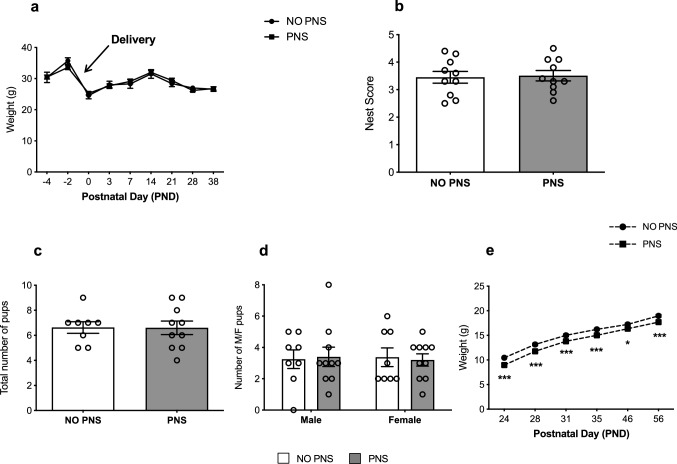
Impact of stress exposure on dams and female offspring. **a** Bodyweight of pregnant control (NO PNS) and stressed (PNS) dams measured during gestational stress and up to 38 days after delivery. **b** Score of the complexity of the nests built by control and stressed mothers and assessed the day after delivery. **c** Total number of pups and **d** number of male and female pups born from control and stressed mothers. **e** Bodyweight of female pups born from control and stressed mothers monitored from PND 24 to PND 56. For all the analyses the data are expressed as mean of the examined variable ± SEM of *n* = 10 mice/group. *P < 0.05, ***P < 0.001 vs. NO PNS (Two-way RM ANOVA with Fisher’s protected LSD)

To establish whether restraint stress exposure affected proper gestation course, starting from GD17 until the 38^th^ day post-partum, we monitored the body weight of control and stressed pregnant mice as well as their capability to build a nest, using the nest test as an indicator of animal well-fare. As shown in Fig. 2, no difference in the body-weight profile (Fig. 2a) or in the nest complexity (Fig. 2b) was found between stressed and control dams. Likewise, we did not observe significant changes in the number of pups per litter or in the sex ratio (Fig. 2c, d).

Subsequently, to establish the lasting effects of prenatal stress (PNS) in the offspring after weaning, we monitored the weight of female pups from PND 24 to PND 56, finding that early stress reduced the body weight of female pups born from stressed dams compared to the non-stressed counterpart (Two-way RM ANOVA, PNS effect: *F*_1,56_ = 25.78, *P* < 0.0001; Fig. 2e).

### Long-Term Effect of Prenatal Stress Exposure on EAE Clinical Signs and Course

**Fig. 3:**
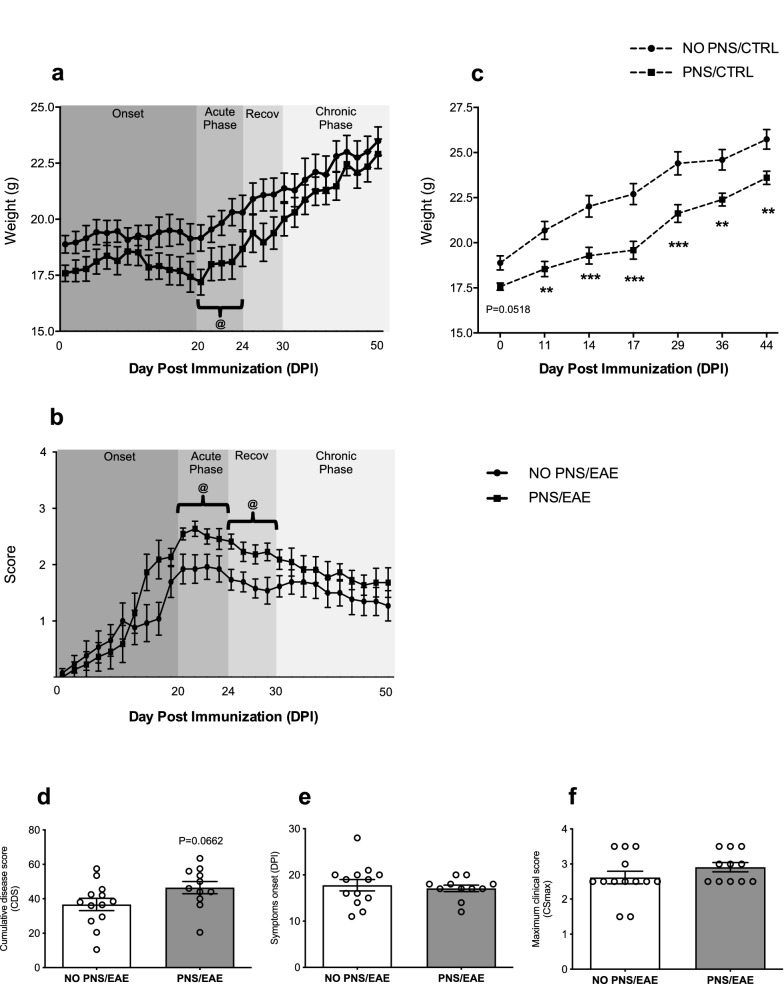
Long-term effect of prenatal stress exposure on EAE clinical signs and course. **a** Body weight and **b** clinical score of EAE animals monitored from DPI 0 to DPI 50 throughout the 4 phases of EAE course (onset, acute phase, recovery, chronic phase). **c** Body weight of non-immunized control and stressed animals from DPI 0 to DPI 44. **d** Cumulative disease score CDS, **e** day of EAE symptoms onset and **f** maximum score, CS max in prenatally stressed and non-stressed EAE mice. For all the analyses the data are expressed as mean of the examined variable ± SEM of *n* = 12 mice/group. **P < 0.01, ***P < 0.001 vs. NO PNS/CTRL; ^@^*P* < 0.05 vs. NO PNS/EAE (Two-way RM ANOVA with Fisher’s protected LSD)

To assess whether PNS could affect EAE course and severity, EAE female pups were daily weighted and scored using the 0–5 grading system for clinical assessment (Furlan et al. 2009) from day post-immunization (DPI) 1 to DPI 50. The clinical profile of EAE symptoms is reported in Fig. 3, where the body weight changes (Fig. 3a) and disability score (Fig. 3b) during the four phases of EAE progression—onset, acute phase, recovery and chronic phase—are shown. The Two-way RM ANOVA analysis indicated that pups born from stressed dams weighed less than their non-stressed counterpart in the acute phase of EAE progression (Two-way RM ANOVA, PNS effect: *F*_1,26_ = 4.979, *P* = 0.0345). It is noteworthy that PNS also reduced the body weight of non-immunized control mice throughout adulthood (Two-way RM ANOVA, PNS effect: *F*_1,25_ = 15.05 *P* = 0.0007; Fig. 3c). Furthermore, clinical manifestations of EAE in prenatally stressed mice were enhanced as compared to the control EAE group as displayed by the increased EAE score over time, especially during the acute phase (Two-way RM ANOVA, PNS effect: *F*_1,22_ = 4.630, *P* = 0.0427) and the following recovery phase (Two-way RM ANOVA, PNS effect: *F*_1,22_ = 7.667, *P* = 0.0112). The cumulative disease score (CDS) was also greater in the PNS/EAE group (Unpaired *t* test: *t*_22_ = 1.933, *P* = 0.0662, Not Significant; Fig. 3d) whereas no difference was observed in the day of onset and in the maximum score (CSmax) (Fig. 3e, f). Taken together, these behavioral results provide clear evidences that PNS enhances the susceptibility to EAE progression in the female offspring.

### Molecular Analyses

#### Analyses of GPR17 and MAG in the Spinal Cord

To gain insight into potential molecular alterations underlying the exacerbated EAE symptomatology observed in mice prenatally exposed to stress, we performed targeted molecular analyses in the cervical portion of the spinal cord of mice at DPI 50. We first analyzed two markers of different stages of oligodendrocytes maturation in the cervical spinal cord: GPR17, a receptor expressed by early oligodendrocyte precursor cells (OPCs) and a key modulator of OPC maturation and myelination (Fumagalli et al. 2015) and MAG, a myelin-associated glycoprotein expressed by mature and differentiated oligodendrocytes.

**Fig. 4:**
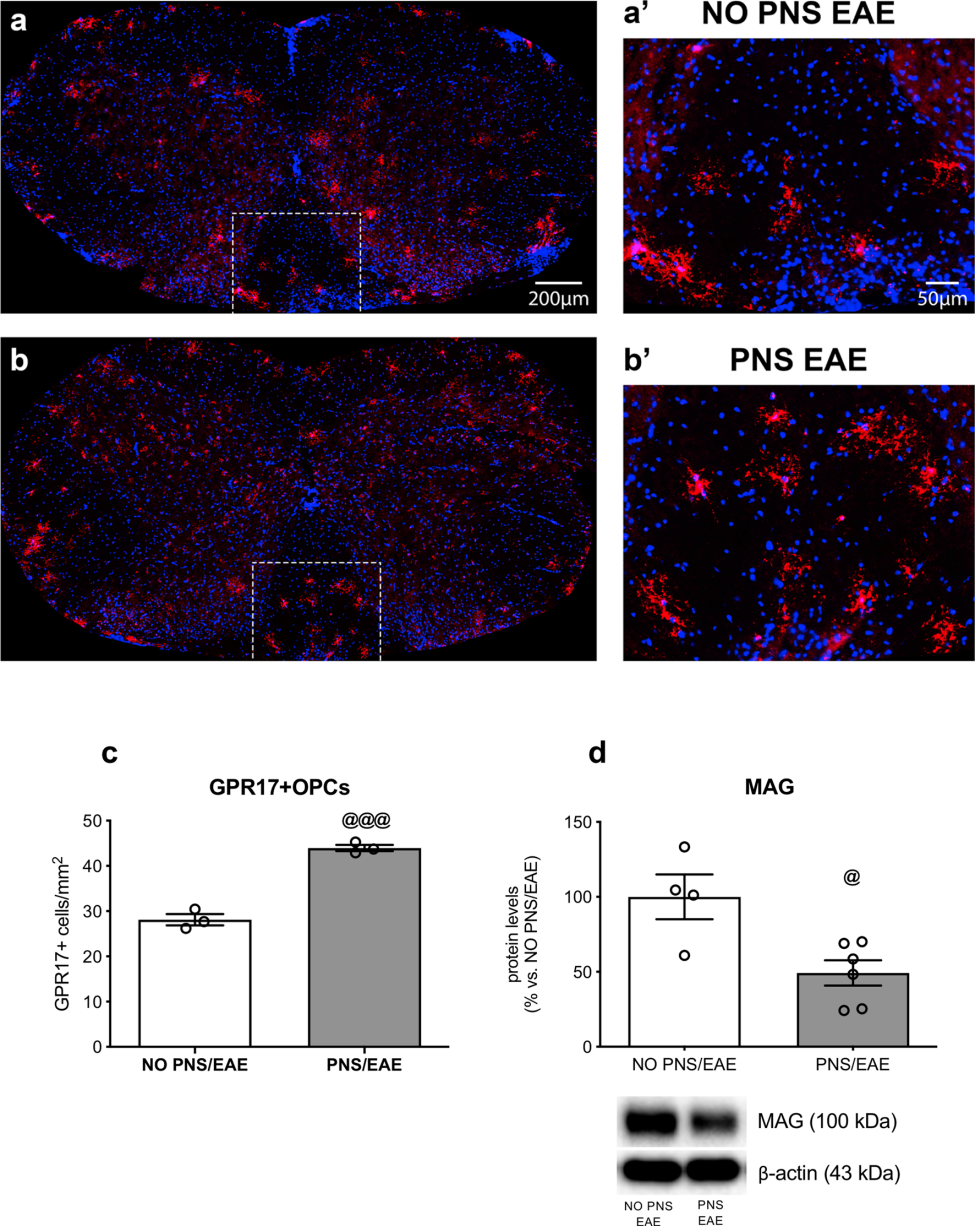
Analyses of GPR17 and MAG in the cervical spinal cord. **a**, **b** Distribution of GPR17 + cells (in red) in cervical spinal cord sections of not stressed mice the developed the encephalomyelitis (NO PNS/EAE) and prenatally stressed animals that developed the encephalomyelitis (PNS/EAE). Cell nuclei were labeled with Hoechst 33258 (in blue). **a**′, **b**′ Higher magnification insets showing GPR17 + cell density in the white matter ventral region. **c** The graph shows the number of GPR17 positive cells per mm^2^ (GPR17 +) in the 2 groups. Cells were counted in the whole white matter of 3 cervical spinal cord sections for each animal (*n* = 3 mice/group). **d** MAG protein levels were investigated in the same region using western blot analysis (*n* = 5/6 mice/group). GPR17 + counting data are expressed as mean of the examined variable ± SEM. MAG protein levels are expressed as a percentage of non-stressed EAE mice (NO PNS/EAE, set at 100%) and represent the mean ± SEM. ^@^*P* < 0.05, ^@@@^*P* < 0.001 vs. NO PNS/EAE (Unpaired *t* test)

The count of GPR17 positive cells (GPR17 +) in the whole white matter of the cervical spinal cord highlighted a greater number of OPCs in EAE animals prenatally exposed to stress compared to non-stressed EAE mice (Unpaired *t* test: *t*_4_ = 11.13; + 57% vs. NO PNS/EAE, *P* = 0.0004), as summarized in Fig. 4, showing reconstructed spinal cord sections of NO PNS/EAE and PNS/EAE mice (Fig. 4a and b), higher magnification insets of GPR17 + cell morphology (Fig. 4a’ and b’) and the numbers of GPR17 positive cells per mm^2^ (Fig. 4c). Consistently, immunoblot results showed a significant decrease of MAG only in stressed EAE animals (Unpaired *t* test: *t*_7_ = 2.309; − 40% vs. NO PNS/EAE, *P* = 0.05; Fig. 4d, Supplementary Fig. 1). Normality and equal variances assumptions were not violated (GPR17 + cells count: Shapiro–Wilk test: *P* > 0.05; *F* test: *F*_2,2_ = 3.249, *P* = 0.4707; MAG immunoblot: Shapiro–Wilk test: *P* > 0.05; *F* test: *F*_3,5_ = 2.094, *P* = 0.4394). Although these data may represent explorative results, given the small sample size, they suggest a defect in oligodendrocyte maturation that potentially leads to impaired remyelination.

#### Analysis of BDNF/Akt/mTOR Signaling

Among the several players involved in the complex process of OPCs differentiation into mature oligodendrocytes, the Akt/mTOR pathway, which can be activated by growth factors, is known to promote differentiation and myelination (Tyler et al. 2009). Indeed, the mammalian target of rapamycin (mTOR), the major downstream player in this pathway, has been implicated in oligodendrocyte differentiation, myelin protein expression, and myelination (Guardiola-Diaz et al. 2012) and is an upstream regulator of GPR17 (Fumagalli et al. 2015; Tyler et al. 2009, 2011; Guardiola-Diaz et al. 2012). To elucidate the role of this pathway in the exacerbated EAE outcome in PNS mice, western blot analyses were performed in the spinal cord using antibodies raised against total and phosphorylated forms of mTOR and Akt.

**Fig. 5:**
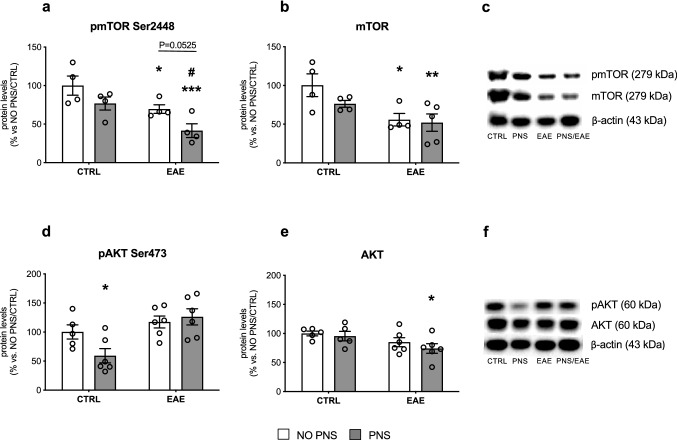
Analysis of BDNF/Akt/mTOR signaling. Protein levels of the kinases phosphor-mTOR at Ser2448 (**a**), mTOR total form (**b**), phospho-AKT at Ser473 (**d**) and AKT total form (**e**) measured in the cervical spinal cord of control (CTRL) or immunized mice that developed the encephalomyelitis (EAE), prenatally exposed to stress (PNS) or not (NO PNS). Representative western blot bands of pmTOR/mTOR (**c**) and pAKT/AKT (**f**). The data, expressed as a percentage of non-stressed CTRL animals (NO PNS/CTRL, set at 100%), represent the mean ± SEM of at least *n* = 4 mice/group. *P < 0.05, **P < 0.01 vs. NO PNS/CTRL; ^#^*P* < 0.05 vs. PNS/CTRL (Two-way ANOVA with Fisher’s protected LSD)

The statistical analyses of Ser2448-phosphorylated-mTOR revealed a significant effect of both PNS (Two-way ANOVA, *F*_1,12_ = 7.758, *P* = 0.0165) and EAE (Two-way ANOVA, *F*_1,12_ = 12.77, *P* = 0.0038). More in detail, as shown in Fig. 5, we observed a significant decrease of phosphorylated-mTOR in EAE mice (− 30% vs. NO PNS/CTRL, *P* = 0.0371) that appeared to be exacerbated in mice previously exposed to PNS (− 58% vs. NO PNS/CTRL, *P* = 0.0007; − 46% vs. PNS/CTRL, *P* = 0.019; Fig. 5a, Supplementary Fig. 2). The Two-way ANOVA analysis of the total form of mTOR showed a significant effect of EAE (*F*_1,13_ = 10.56, *P* = 0.0063) that results in a broad reduction trend in all EAE mice (NO PNS: − 44% vs. NO PNS/CTRL, *P* = 0.0124; PNS: − 48% vs. NO PNS/CTRL, *P* = 0.0056; Fig. 5b, Supplementary Fig. 2). Normality and equal variances assumptions were not violated (pmTOR: Shapiro–Wilk test: *P* > 0.05; Brown-Forsythe test: *F*_3,12_ = 1.045, *P* = 0.4080; mTOR: Shapiro–Wilk test: *P* > 0.05; Brown-Forsythe test: *F*_3,13_ = 1.720, *P* = 0.2121).These explorative results suggest that intrauterine stress exposure had a negative impact on mTOR activation in EAE animals.

Next, we analyzed the levels of the protein kinase Akt which is known to stimulate mTOR activity (Dibble and Cantley 2015). The statistical analyses of Serine 473 phosphorylated form of Akt revealed a significant effect of EAE (Two-way ANOVA, *F*_*1*,19_ = 11.62, *P* = 0.0029). However, multiple comparisons analyses depicted a significant decrease due to PNS exposure alone (− 41% vs. NO PNS/CTRL, *P* = 0.0331) and we did not observe any specific modulation of phospho-Akt in EAE animals compared to NO PNS/CTRL mice (Fig. 5d, Supplementary Fig. 3). On the contrary, in line with our previous observations, we detected a significant effect of EAE (Two-way ANOVA, *F*_1,1_ = 5.880, *P* = 0.0261) that resulted in a reduction of the total form of the protein only in PNS/EAE animals (− 26% vs. NO PNS/CTRL, *P* = 0.025; Fig. 5e, Supplementary Fig. 3). Normality and equal variances assumptions were not violated (pAkt: Shapiro–Wilk test: *P* > 0.05; Brown-Forsythe test: *F*_3,19_ = 0.3192, *P* = 0.8114; Akt: Shapiro–Wilk test: *P* > 0.05; Brown-Forsythe test: *F*_3,18_ = 0.4931, *P* = 0.6915). Overall, our results confirm the implication of Akt/mTOR pathway in the impaired neurological score of these animals.

#### Analysis of BDNF Protein Levels

**Fig. 6:**
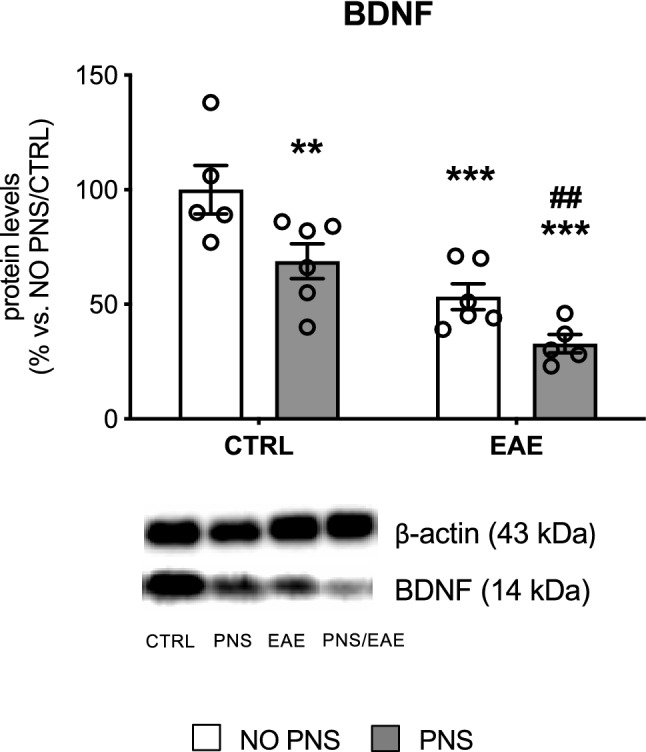
Analysis of BDNF protein levels.The protein levels of the mature form of the neurotrophin BDNF were measured in the cervical spinal cord of control (CTRL) or immunized mice that developed the encephalomyelitis (EAE), prenatally exposed to stress (PNS) or not (NO PNS). The data, expressed as a percentage of non-stressed CTRL animals (NO PNS/CTRL, set at 100%), represent the mean ± SEM of at least *n* = 5 mice/group.*P < 0.05, **P < 0.01 vs. NO PNS/CTRL; ^#^*P* < 0.05 vs. PNS/CTRL (Two-way ANOVA with Fisher’s protected LSD)

The Akt-mTOR pathway is one of the three main signaling cascades triggered by the binding of the neurotrophin BDNF to its high-affinity tropomyosin receptor kinase B (TrkB), alongside the mitogen-activated protein kinase (MAPK) and the phospholipase Cγ (PLCγ) pathways (Numakawa et al. 2010). Given the role of BDNF in the maintenance of neural plasticity and stress-related diseases, we assessed the protein levels of its mature form in the whole homogenate prepared from the cervical portion of the spinal cord of the mice. We found a significant PNS (Two-way ANOVA, *F*_1,18_ = 12.53, *P* = 0.0023) and EAE effect (Two-way ANOVA, *F*_1,18_ = 32.07, *P* < 0.0001). Indeed, as displayed in Fig. 6 (Supplementary Fig. 4), BDNF levels were significantly reduced in PNS mice (− 31% vs. NO PNS/CTRL, *P* = 0.0074) as well as in EAE animals (− 47% vs. NO PNS/CTRL, *P* = 0.0003). Interestingly, the decrease was exacerbated in EAE mice prenatally exposed to stress (− 67% vs. NO PNS/CTRL, *P* < 0.0001, − 52% vs. PNS/CTRL, *P* = 0.0026). Normality and equal variances assumptions were not violated (Shapiro–Wilk test: *P* > 0.05; Brown-Forsythe test: *F*_3,18_ = 0.6694, *P* = 0.5818).

#### Analysis of BDNF Gene Expression

Next, we evaluated if our experimental paradigm could also affect BDNF gene expression. BDNF gene has a complex structure consisting of eight 5′ untranslated exons alternatively spliced to a common 3′ exon (exon IX), which contains the protein coding region. Moreover, two alternative polyadenylated transcription stop sites are located within the common 3′ exon IX, which generate two distinct pools of mRNA with either short or long 3′untranslated regions (3′-UTRs). It is known that BDNF transcripts characterized by short 3′-UTR are restricted to the soma, whereas long 3′-UTR mRNAs are mainly localized in dendrites (An et al. 2008). Accordingly, we assessed by Real Time RT-PCR the mRNA levels of total BDNF (transcript IX), the mRNA levels of BDNF marked by long 3′-UTR as well as two major splice variants, isoforms IV and VI.

**Fig. 7:**
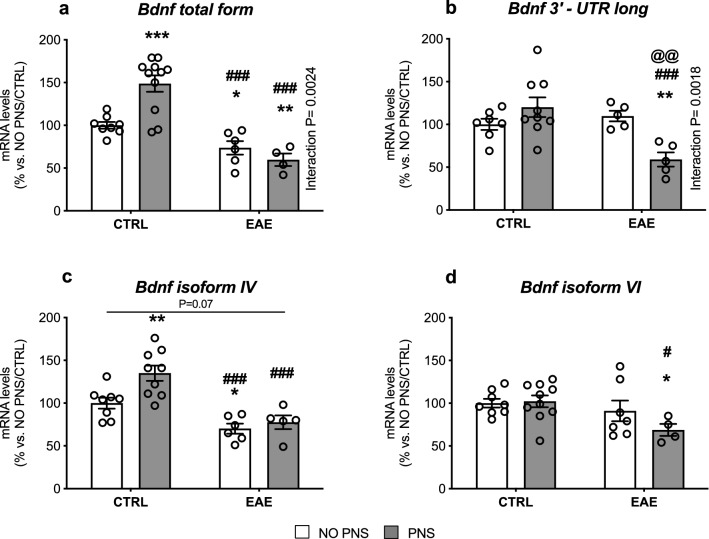
Analysis of BDNF gene expression. The mRNA levels of BDNF total form (**a**), 3′-UTR long form (**b**), isoform IV (**c**) and isoform VI (**d**) were measured in the cervical spinal cord of control (CTRL) or immunized mice that developed the encephalomyelitis (EAE), prenatally exposed to stress (PNS) or not (NO PNS). The data, expressed as a percentage of non-stressed CTRL animals (NO PNS/CTRL, set at 100%), represent the mean ± SEM of at least *n* = 5 mice/group. *P* < 0.05, **P < 0.01, ***P < 0.001 vs. NO PNS/CTRL; ^#^*P* < 0.05, ^###^*P* < 0.001 vs. PNS/CTRL; ^@@^*P* < 0.01 vs. NO PNS/EAE (Two-way ANOVA with Fisher’s protected LSD)

We found that the gene expression of total BDNF was significantly modulated by EAE (Two-way ANOVA, *F*_1,25_ = 38.67, *P* < 0.0001) and by a significant PNS x EAE interaction (Two-way ANOVA *F*_1,25_ = 11.43, *P* = 0.0024). As a result, the mRNA levels of total BDNF (Fig. 7a) were up-regulated in animals prenatally exposed to stress (+ 49% vs. NO PNS/CTRL, *P* = 0.0001) and reduced by the encephalomyelitis (− 26% vs. NO PNS/CTRL, *P* = 0.047) an effect even higher in PNS/EAE animals (− 40% vs. NO PNS/CTRL, *P* = 0.0093; − 60% vs. PNS/CTRL, *P* < 0.0001). A partially different profile was observed for the gene expression of the long 3′-UTR pool of transcripts (Fig. 7b). Indeed, we found a significant EAE effect (Two-way ANOVA, *F*_1,22_ = 6.623, *P* = 0.0172) and PNS x EAE interaction (Two-way ANOVA, *F*_1,22_ = 12.53, *P* = 0.0018) displayed by a strong reduction of long 3′-UTR BDNF only in PNS/EAE mice compared to control mice (− 41% vs. NO PNS/CTRL, *P* = 0.0095), to prenatally stressed mice (− 50% vs. PNS/CTRL, *P* = 0.0002) and to EAE animals not exposed to stress (− 46% vs. NO PNS/EAE, *P* = 0.0037). The analysis of isoform IV gene expression displayed a significant EAE (Two-way ANOVA, *F*_1,24_ = 29.27, *P* < 0.0001) and PNS (Two-way ANOVA, *F*_1,24_ = 6.925, *P* = 0.0146) effect. Similar to what was observed for the total form of the neurotrophin, we found an increase in PNS mice (+ 35% vs. NO PNS/CTRL, *P* = 0.002) paralleled by a decrease after EAE in both non stressed (− 30% vs. NO PNS/CTRL, *P* = 0.0137; − 48% vs. PNS/CTRL, *P* < 0.0001) and prenatally stressed mice (− 22% vs. NO PNS/CTRL, *P* = 0.07, Not Significant; − 42% vs. PNS/CTRL, *P* < 0.0001; Fig. 7c). Conversely, the modulation of isoform VI was similar to what was observed for the long 3′ UTR BDNF, with a significant effect of EAE (Two-way ANOVA, *F*_1,25_ = 5.846, *P* = 0.0232) that resulted in a decrease only in the EAE mice prenatally exposed to stress (− 31% vs. NO PNS/CTRL, *P* = 0.0311; − 32% vs. PNS/CTRL, *P* < 0.0180; Fig. 7d). Normality and equal variances assumptions were not violated (BDNT tot: Shapiro–Wilk test: *P* > 0.05; Brown-Forsythe test: *F*_3,25_ = 1.056, *P* = 0.3852; BDNF 3′-UTR long: Shapiro–Wilk test: *P* > 0.05; Brown-Forsythe test: *F*_3,22_ = 0.9451, *P* = 0.4358; Isoform IV: Shapiro–Wilk test: *P* > 0.05; Brown-Forsythe test: *F*_3,24_ = 1.264, *P* = 0.3091; Isoform VI: Shapiro–Wilk test: *P* > 0.05; Brown-Forsythe test: *F*_3,25_ = 1.016, *P* = 0.4022).

## Discussion

It is well known that adverse events in utero can markedly affect neurodevelopment and induce profound long-lasting alterations in offspring, influencing maturational trajectories and leading to long-lasting alterations eventually resulting in enhanced susceptibility to several diseases later in life (Coe and Lubach 2005). Indeed, this “fetal programming” hypothesis, by which an insult occurring in a critical period of development has lasting effects (Barker 1998), has been found to be relevant for metabolic disorders such as obesity and metabolic syndrome (Lau et al. 2011), cardiovascular diseases (Alexander et al. 2015), neuropsychiatric and neurodegenerative disorders (Faa et al. 2016; Modgil et al. 2014). Among this plethora of diseases characterized by susceptibility to fetal programming, little is known about the impact of insults happening to the intrauterine life on multiple sclerosis (MS). While several preclinical studies have been carried out on the effects of environmental risk factors on MS during adulthood (Krementsov and Teuscher 2013), less is known on the influence of developmental stress exposure and only a few studies have addressed this gap by evaluating the impact of earlier post-natal events -such as neonatal handling and cross-fostering- in the experimental autoimmune encephalomyelitis (EAE) model of MS in rats (Laban et al. 1995; Dimitrijević et al. 1994) or in mice (Columba-Cabezas et al. 2009; Case et al. 2010).To our knowledge, the only scientific evidence on insults occurring specifically during pregnancy concerns the consequences of maternal infection on EAE course in the offspring (Solati et al. 2012; Majidi-Zolbanin et al. 2015). The behavioral and molecular effects of gestational exposure to stress on EAE course have not been investigated, and our work indeed represents the first study aiming to address this aspect.

Here we demonstrated that maternal stress during the last days of gestation worsens EAE outcome in the adult female offspring. Although our stress protocol has two potential confounding aspects—the potential additional stress load due to mice shipment at GD14 and bystander stress experienced by control dams—that may disguise the impact of the restraint stress paradigm, we identified a long-lasting phenotype in the female offspring born from stressed dams. Specifically, despite no differences in the weight or in nest building abilities were found between control and stressed mothers, we proved that gestational stress exerts long-lasting effects on the offspring by decreasing the body weight of the progeny and by increasing susceptibility to EAE as indicated by the more severe symptoms scoring of prenatally stressed animals, which was statistically significant in both the acute and recovery phases of EAE course. While weight reduction is a well-established effect of prenatal stress exposure (Guan et al. 2016; Brunton 2013), to our knowledge this is the first evidence concerning the impact of stress in utero on EAE outcome. We believe that the EAE exacerbation observed in our study could not be attributed only to the weight lowering induced by PNS. Indeed, in the present work we proposed a potential mechanism that could explain the behavioral outcome independently from the weight, as our molecular analyses were performed at DPI 50, when the body weight of the two groups was no longer statistically different. Moreover, our behavioral results are in line with other studies reporting stress-related exacerbation of the disease at both behavioral and molecular levels. It has been demonstrated that chronic restraint stress during adulthood enhances demyelination in another MS animal model, the Theiler’s murine encephalomyelitis virus (TMEV) infection (Young et al. 2013) and that chronic sound stress resulted in increased severity of neurological signs and histological lesions of the spinal cord in stressed EAE rats compared to the non-stressed ones (Núñez-Iglesias et al. 2010). Moreover, acute immobilization stress in adult animals shortens the time to EAE onset (Chandler et al. 2002).

Previous studies have shown that the EAE acute phase is characterized by an increased number of GPR17-expressing cells blocked at immature stages and therefore no longer contributing to remyelination (Chen et al. 2009; Coppolino et al. 2018). Our data suggest that PNS exacerbates this defect, and that the stress-induced EAE severity may be due, at least in part, to an impaired remyelination process in the spinal cord, where more GPR17-positive immature oligodendrocytes were found. This effect could have important translational relevance since GPR17, a G protein-coupled receptor which is physiologically down-regulated after the immature oligodendrocyte stage, has been identified as an ideal target for new regenerative therapeutic approaches for MS and other myelin-associated disorders (Fancy et al. 2010; Fumagalli et al. 2017; Lu et al. 2018). To further investigate the potential mechanisms underlying its modulation by prenatal stress, we focused our analyses on Akt/mTOR signaling since mTOR has a pivotal role in cell growth, differentiation and survival, and has been implicated in oligodendrocyte development and myelination as well as in GPR17 regulation (Fumagalli et al. 2015; Tyler et al. 2009). Moreover, preclinical studies indicate that, at cerebral level, this pathway is influenced by different stress paradigms (Chandran et al. 2013; Xia et al. 2016). In line with our hypothesis, despite the small sample size, we observed a decrease of phosphorylated levels of mTOR as well as of the total form of Akt, the protein kinase known to stimulate mTOR activation. It is worth mentioning that the modulation of GPR17 by mTOR observed here is supported by results from a proteomic analysis revealing an increase of GPR17 in OPCs cultures treated with the mTOR inhibitor rapamycin (Tyler et al. 2011), an effect likely due to reduction of the G protein-coupled receptor kinase (GRK2) that could, in turn, prevent physiological GPR17 down-regulation via the key regulator of cell proliferation and apoptosis Murine Double Minute2 (MDM2) (Fumagalli et al. 2015). Interestingly, the Akt/mTOR pathway is known to be activated by several growth factors, including the neurotrophin Brain-derived neurotrophic factor (BDNF) (Yoshii and Constantine-Paton 2010). The link between stress and BDNF is well-established (Molteni et al. 2016; Gray et al. 2013; McEwen et al. 2015; Calabrese et al. 2014), and different studies show that prenatal stress exposure can lead to BDNF alterations later in adulthood, both in the brain (Boersma et al. 2014; Blaze et al. 2017; Yeh et al. 2012; Luoni et al. 2014a, 2014b) and in the spinal cord (Winston et al. 2014). Furthermore, BDNF has a crucial role in cell growth and survival (Murray and Holmes 2011) and several studies support the hypothesis of a neuroprotective function of this neurotrophin in myelination and myelin repair (Acosta et al. 2013; De Santi et al. 2009; Linker et al. 2010). Consistently, BDNF treatment using transformed bone marrow stem cells reduces inflammation and apoptosis in EAE mice (Makar et al. 2008) and delays symptoms onset, reducing the overall EAE clinical severity (Makar et al. 2009).

It is important to note that, in our study, the modulation of the Akt/mTOR pathway and the consequent increase in the number of GPR17-positive OPCs we observed in prenatally stressed EAE mice are paralleled by a reduction of BDNF. Specifically, the neurotrophin protein levels were reduced in the spinal cord of all mice subjected to EAE induction similarly to what was observed for total BDNF mRNA levels, while the gene expression of the long 3′ UTR BDNF form was down-regulated only in prenatally stressed mice. This form of BDNF represents the pool of transcripts localized at dendritic level due to the “dendritic targeting” process (Tongiorgi 2008), which occurs in an activity-dependent manner and enables the local synthesis of proteins required for neuronal development and plasticity, two features known to be altered by stress exposure (Wang et al. 2017). In line with this profile, we observed a similar modulation for BDNF isoform VI which belongs to this pool of transcripts and is localized in the distal dendrites (Baj et al. 2011). On the contrary isoform IV, spatially segregated in the soma/proximal portion of the dendrite, was broadly decreased in all EAE mice, suggesting that PNS may alter the proper dendritic targeting of BDNF transcripts, thus leading to a cascade of molecular events culminating in impaired maturation of OPCs and in more severe EAE symptomatology.

In conclusion, our study demonstrates for the first time that stressful events occurring during the intrauterine life may exacerbate EAE clinical manifestations in the female population, giving new insights in the role of early life adversities in the etiopathogenesis of EAE/MS. Our data also suggest that PNS affects oligodendrocytes maturation in the spinal cord through an impairment of the AKT/mTOR pathway associated with a reduction of BDNF levels. Since several already marketed drugs are able to modulate BDNF levels, the possibility of drug repositioning for multiple sclerosis should be addressed in future studies.

## Electronic supplementary material

Below is the link to the electronic supplementary material.

Supplementary file1 (PPTX 2827 KB)

## Data Availability

All data generated or analyzed during this study are included in this published article.

## Abbreviations

Akt: Protein kinase B
BDNF: Brain-derived neurotrophic factor
DPI: Days post injection
EAE: Experimental autoimmune encephalomyelitis
GPR17: G Protein-Coupled Receptor 17
GD: Gestational day
mTOR: Mammalian target of rapamycin complex 1
MS: Multiple sclerosis
MAG: Myelin-associated glycoprotein
MOG: Myelin oligodendrocyte glycoprotein
OPCs: Oligodendrocytes precursor cells
PTX: Pertussis toxin
PND: Postnatal day
PNS: Prenatal stress
