# Supplementary material for: Prenatal Stress Impairs Spinal Cord Oligodendrocyte Maturation via BDNF Signaling in the Experimental Autoimmune Encephalomyelitis Model of Multiple Sclerosis
Source: Cell Mol Neurobiol. 2020 Dec 1;42(4):1225–40. doi: 10.1007/s10571-020-01014-x (PMC8942968; doi:10.1007/s10571-020-01014-x)
Supplement: Supplementary file 1 — Supplementary file1 (PPTX 2827 KB) [file 10571_2020_1014_MOESM1_ESM.pptx]

## Slide 1
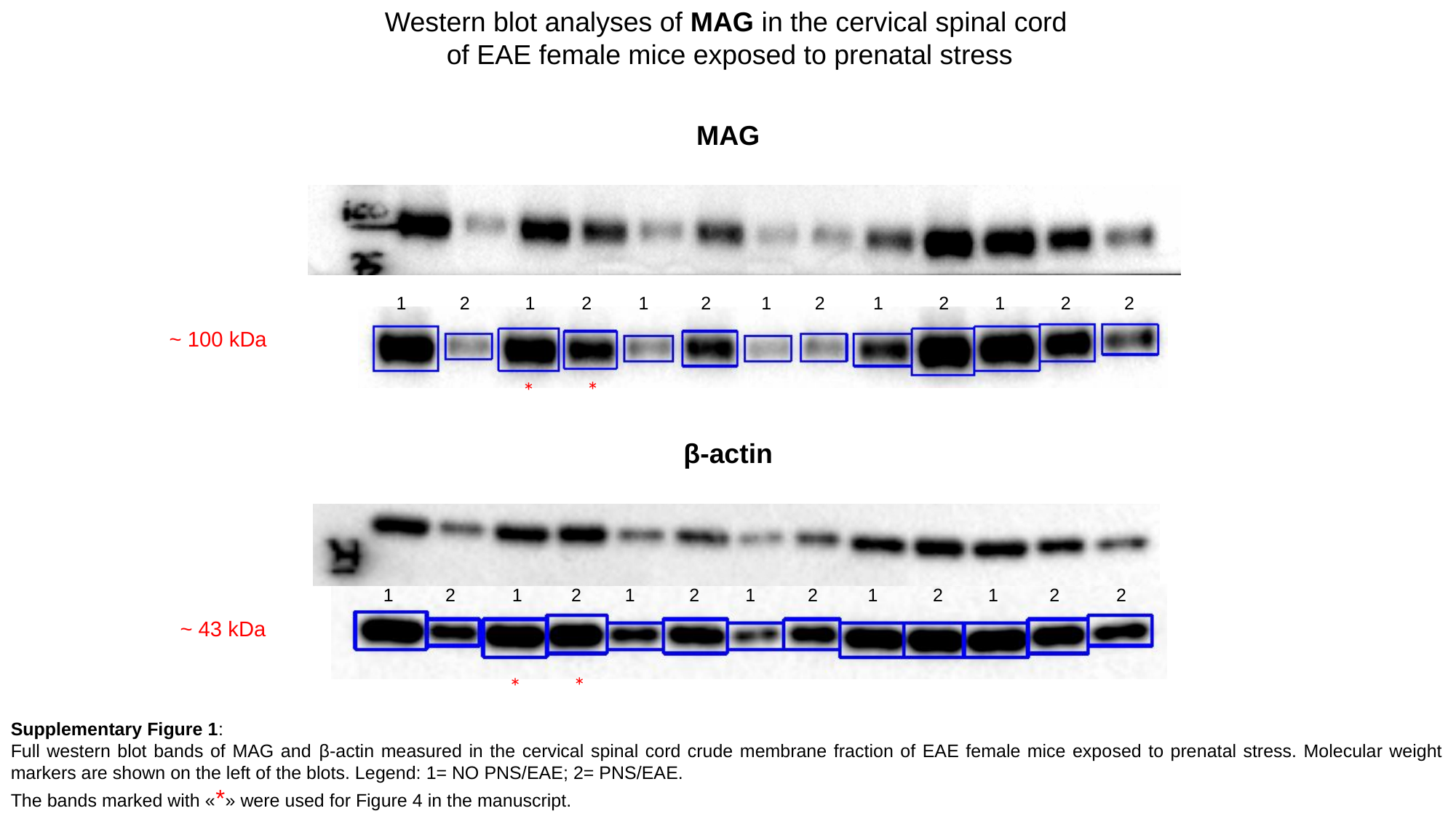

Western blot analyses of MAG in the cervical spinal cord
of EAE female mice exposed to prenatal stress
MAG
1
2
1
2
1
2
1
2
1
2
1
2
2
~ 100 kDa
*
*
β-actin
1
2
1
2
1
2
1
2
1
2
1
2
2
~ 43 kDa
*
*
Supplementary Figure 1:
Full western blot bands of MAG and β-actin measured in the cervical spinal cord crude membrane fraction of EAE female mice exposed to prenatal stress. Molecular weight markers are shown on the left of the blots. Legend: 1= NO PNS/EAE; 2= PNS/EAE.
The bands marked with «*» were used for Figure 4 in the manuscript.

## Slide 2
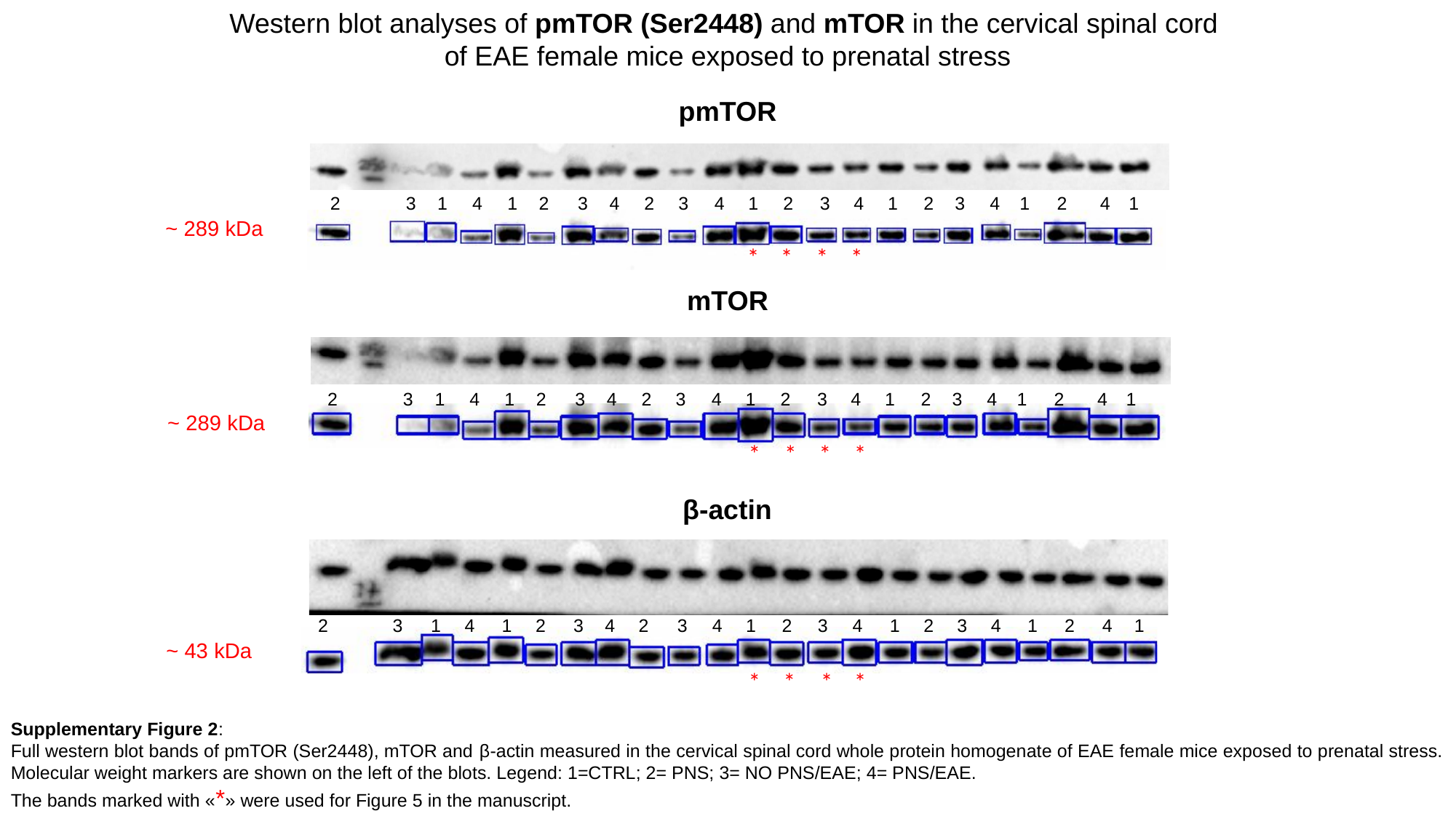

Western blot analyses of pmTOR (Ser2448) and mTOR in the cervical spinal cord
of EAE female mice exposed to prenatal stress
pmTOR
2
3
1
4
1
2
3
4
2
3
4
1
2
3
4
1
2
3
4
1
2
4
1
~ 289 kDa
*
*
*
*
mTOR
2
3
1
4
1
2
3
4
2
3
4
1
2
3
4
1
2
3
4
1
2
4
1
~ 289 kDa
*
*
*
*
β-actin
2
3
1
4
1
2
3
4
2
3
4
1
2
3
4
1
2
3
4
1
2
4
1
~ 43 kDa
*
*
*
*
Supplementary Figure 2:
Full western blot bands of pmTOR (Ser2448), mTOR and β-actin measured in the cervical spinal cord whole protein homogenate of EAE female mice exposed to prenatal stress. Molecular weight markers are shown on the left of the blots. Legend: 1=CTRL; 2= PNS; 3= NO PNS/EAE; 4= PNS/EAE.
The bands marked with «*» were used for Figure 5 in the manuscript.

## Slide 3
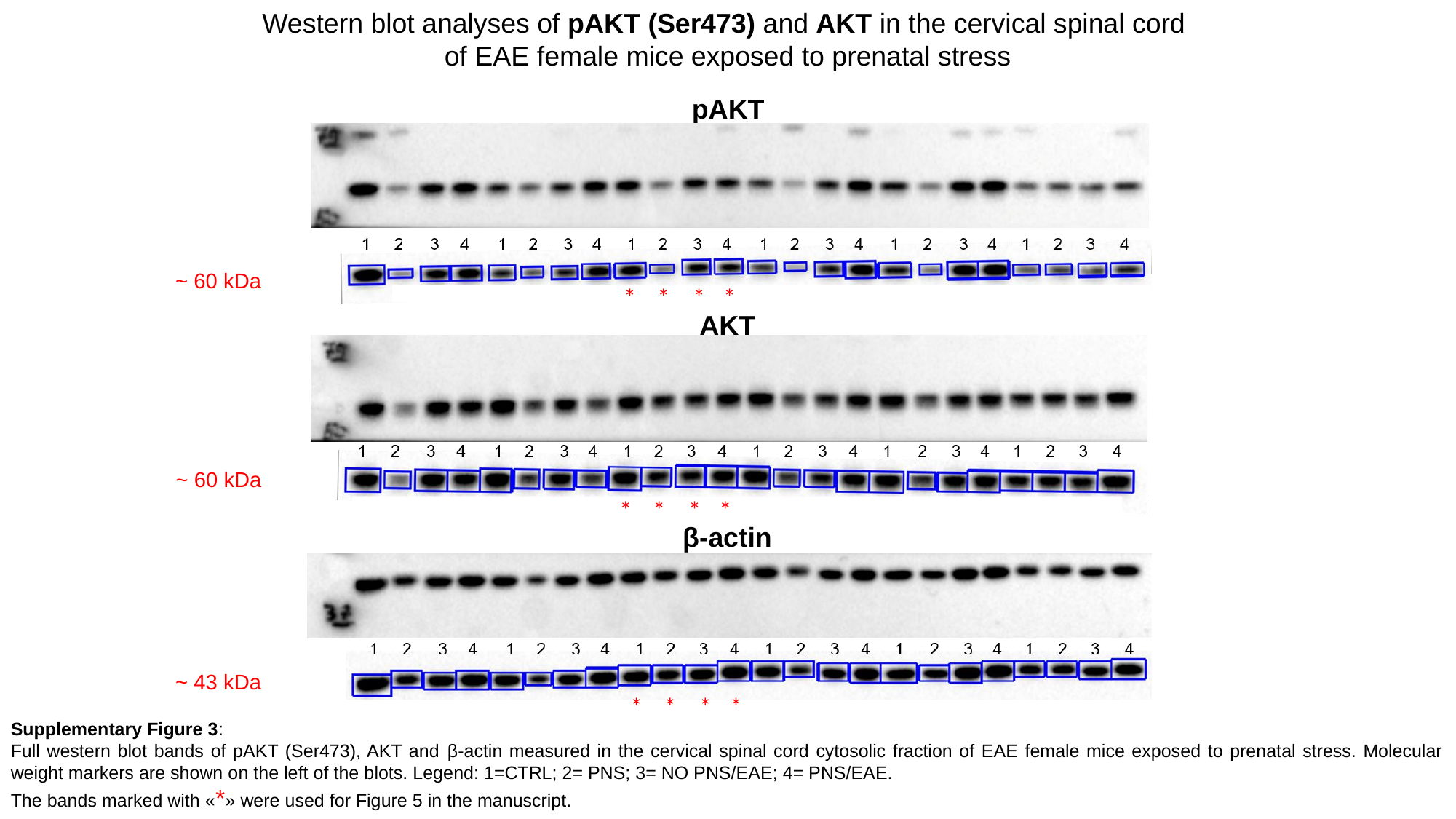

Western blot analyses of pAKT (Ser473) and AKT in the cervical spinal cord
of EAE female mice exposed to prenatal stress
pAKT
~ 60 kDa
*
*
*
*
AKT
~ 60 kDa
*
*
*
*
β-actin
~ 43 kDa
*
*
*
*
Supplementary Figure 3:
Full western blot bands of pAKT (Ser473), AKT and β-actin measured in the cervical spinal cord cytosolic fraction of EAE female mice exposed to prenatal stress. Molecular weight markers are shown on the left of the blots. Legend: 1=CTRL; 2= PNS; 3= NO PNS/EAE; 4= PNS/EAE.
The bands marked with «*» were used for Figure 5 in the manuscript.

## Slide 4
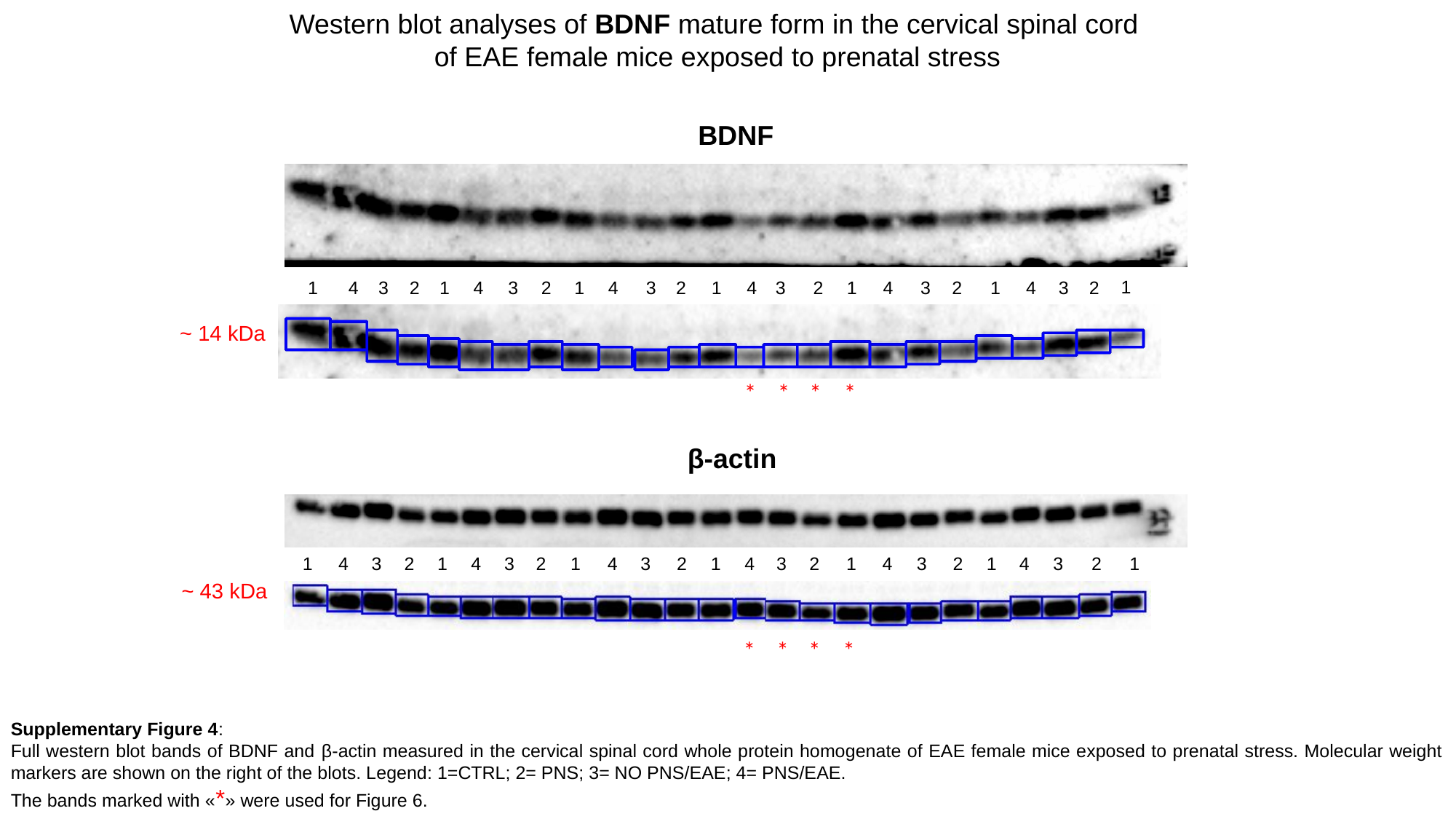

Western blot analyses of BDNF mature form in the cervical spinal cord
of EAE female mice exposed to prenatal stress
BDNF
1
1
4
3
2
1
4
3
2
1
4
3
2
1
4
3
2
1
4
3
2
1
4
3
2
~ 14 kDa
*
*
*
*
β-actin
1
1
4
3
2
1
4
3
2
1
4
3
2
1
4
3
2
1
4
3
2
1
4
3
2
~ 43 kDa
*
*
*
*
Supplementary Figure 4:
Full western blot bands of BDNF and β-actin measured in the cervical spinal cord whole protein homogenate of EAE female mice exposed to prenatal stress. Molecular weight markers are shown on the right of the blots. Legend: 1=CTRL; 2= PNS; 3= NO PNS/EAE; 4= PNS/EAE.
The bands marked with «*» were used for Figure 6.
